# Interplay Between Endocrine Disruptors and Immunity: Implications for Diseases of Autoreactive Etiology

**DOI:** 10.3389/fphar.2021.626107

**Published:** 2021-03-23

**Authors:** Maria Popescu, Talia B. Feldman, Tanuja Chitnis

**Affiliations:** ^1^ Harvard Medical School, Boston, MA, United States; ^2^ Brigham Multiple Sclerosis Center, Department of Neurology, Brigham and Women’s Hospital, Boston, MA, United States; ^3^ Ann Romney Center for Neurologic Diseases, Brigham and Women’s Hospital, Boston, MA, United States

**Keywords:** endocrine disruptors, immunity, autoimmunity, environmental factors, sex hormones

## Abstract

The sex-bias of disease susceptibility has remained a puzzling aspect of several autoimmune conditions, including post-infection viral autoimmunity. In the last half of the twentieth century, the incidence rate of female-biased autoimmunity has steadily increased independent of medical advances. This has suggested a role for environmental factors, such as endocrine disrupting chemicals, which have been described to interfere with endocrine signaling. Endocrine involvement in the proper function of innate and adaptive immunity has also been defined, however, these two areas have rarely been reviewed in correlation. In addition, studies addressing the effects of endocrine disruptors have reported findings resulting from a broad range of exposure doses, schedules and models. This experimental heterogeneity adds confusion and may mislead the translation of findings to human health. Our work will normalize results across experiments and provide a necessary summary relevant to human exposure. Through a novel approach, we describe how different categories of ubiquitously used environmental endocrine disruptors interfere with immune relevant endocrine signaling and contribute to autoimmunity. We hope this review will guide identification of mechanisms and concentration-dependent EDC effects important not only for the sex-bias of autoimmunity, but also for other conditions of immune dysfunction, including post-infection autoreactivity such as may arise following severe acute respiratory syndrome coronavirus 2, Epstein-Barr virus, Herpes Simplex virus.

## Introduction

The gradual increase in the female to male disease susceptibility ratio, including for autoimmune and metabolic disease, has been globally reported despite progressive medical advancements. In parallel, production of synthetic materials and their implementation in commercial goods has seen a similar upward trend. To meet consumer demand, a variety of products have now been manufactured using chemical additives. Several of these synthetic compounds have the ability to disrupt normal endocrine function and have appropriately been termed endocrine-disrupting chemicals (EDCs), or xenohormones. Widespread detection of EDCs in the serum and adipose tissue of humans and wildlife alike has drawn attention to the role of environmental factors. Sex-hormones, most notably estrogen, are established modulators of immune cell populations (Orton, Herrera et al., 2006; Bove and Chitnis 2014). EDCs with estrogenic activity may have therefore contributed to the steady increase in the female to male disease susceptibility ratio, raising concern over their continued ability to modulate immunity in the future. To understand the diversity and sex-bias of immune responses as well as potential triggers for autoimmunity and post-infection autoimmunity, we will provide a comprehensive account of estrogen signaling modalities and the role of estrogen-like EDCs in innate and adaptive immunity.

The choice of focused categories herein presented is relative to the vast diversity of EDCs. The risk for environmental contamination and human exposure depends on the degree and use frequency of EDCs in consumer products. Three such ubiquitous categories are (1) phenols, (2) parabens, and (3) phthalates. Xenoestrogens inclusive of each category exhibit various immunomodulatory functions and are therefore implicit in immune dysfunction, as well as, in the onset and progression of diseases with autoreactive attributes. Together with the inherent dependency of female biology on estrogen, EDCs emerge as driving factors for the increased female to male disease susceptibility ratio. To illustrate, adaptive immune dysfunction induced by phenols results in reduced influenza A viral titers in females, but enhanced production of autoantibodies and overall IgM secretion (Yurino et al., 2004; Peretz et al., 2016). Likewise, parabens are associated with diabetic autoimmunity, such as gestational diabetes and the increased onset risk for future diabetes (Liu et al., 2019). Phthalate compounds and their metabolites similarly enhance insulin resistance and lead to an increased risk of type 1 and 2 diabetes (Castro-Correia et al., 2018; Radke et al., 2019). Furthermore, phenols, parabens and phthalates all have effects upon innate immunity, including on cytokine secretion, which indicates that EDCs inadvertently play an additional role in the dysfunction of adaptive responses.

Despite compelling evidence, studies of EDC effects have reported results across a broad range of exposure doses, schedules and models, which has added heterogeneity and confusion in translating findings to human health. Our review will normalize results across experiments for each EDC category, to provide a summary of effects relevant to innate and adaptive immunity, as well as for autoimmunity. Because EDCs have rarely been reviewed in the context of immunoendocrine interactions, we also describe normal estrogen signaling modalities and the likely influence of the three EDC categories. To the best of our knowledge, this approach will be novel and can expand environmental considerations for different aspects of immunity. We hope this review will guide the identification of mechanisms and exposure effects that are important not only for the sex-bias of autoimmune diseases, but also for related conditions of immune dysfunction, including the post-infection autoreactivity characterized for severe acute respiratory syndrome coronavirus 2 (SARS-CoV-2), Epstein-Barr virus (EBV), Herpes Simplex virus and others (Alexopoulos et al., 2018; Maslinska, 2019; Dalakas, 2020; Lazarian et al., 2020; Mallapaty, 2020).

### Categorization of EDCs

EDCs belong to the broad classification of chemical additives, including plasticizers, resins, and antimicrobial agents. The formal definition for EDCs describes them as, “environmental agents that interfere with the normal function of endogenous hormones”. Exposure results from either the direct use of diverse household commodities, such as personal care products, toys, electronics and others, or from contaminated environmental sources, such as, soil and water (Di Nisio and Foresta, 2019). Toxicological findings have identified effects of EDCs upon critical periods of development, including prenatal, perinatal, and pubertal development (Gorski, 2002). Such early developmental disruptions cause hormonal dysfunction and establish lifelong consequences, such as increased susceptibility to disease (Collman, 2011).

Xenoestrogens are a subset of EDCs that exhibit structural and functional mimicry of estrogen, including phenols, parabens and phthalates. Phenol EDCs include benzene-derived compounds, for which exposure generally occurs through inhalation, ingestion, or dermal contact. In humans, phenol urine levels less than 1,000 μg/L are considered normal (Ong and Lee, 1994). Past reports have indicated that 55% of Americans had detectable 4-tertiary-octylphenol (OP) in urine (0.2–20.6 μg/L) and high bioaccumulation of nonylphenol (NP) in breast milk (56.3 μg/L) (Calafat et al., 2008; Hwang et al., 2015). Levels of bisphenol A (BPA, 0.7–2.3 ug/L), bisphenol S (BPS, 0.2–0.8 ug/L) and bisphenol F (BPF, LOD-0.7 ug/L) have also been reported (Jacobson et al., 2019).

The second category of EDCs, parabens, include alkyl esters of *p*-hydroxybenzoic acid, such as methyl (MP), ethyl (EP), propyl (PP) and butyl (BP) parabens. Exposure occurs primarily through dermal absorption (2,400 μg/kg/d), but also through ingestion of food (13 μg/kg/d) and pharmaceuticals (417 μg/kg/d). Similar with phenols, parabens are also detected in human breast milk (total: 1.87–49 µg/L) (Park et al., 2019; Dualde et al., 2020).

Phthalates, derived from the esterification of phthalic acids, comprise the third EDC category. Akin to phenols and parabens, exposure includes oral, dermal and inhalation pathways. Although phthalates do not generally bioaccumulate, they have low target binding and can leak from products into the environment (Heudorf et al., 2007). Manufacturing changes have led to decreased detection of certain parabens, such as dibutyl phthalate (DBP) and di (2-ethylhexyl) phthalate (DEHP). In contrast, levels of substitute phthalates, including diisobutyl phthalate (DiBP) and diisononyl phthalate (DNP), have increased (Wittassek et al., 2007).

### Estrogen Signaling

#### Estrogen Receptor-α and -β

**FIGURE 1:**
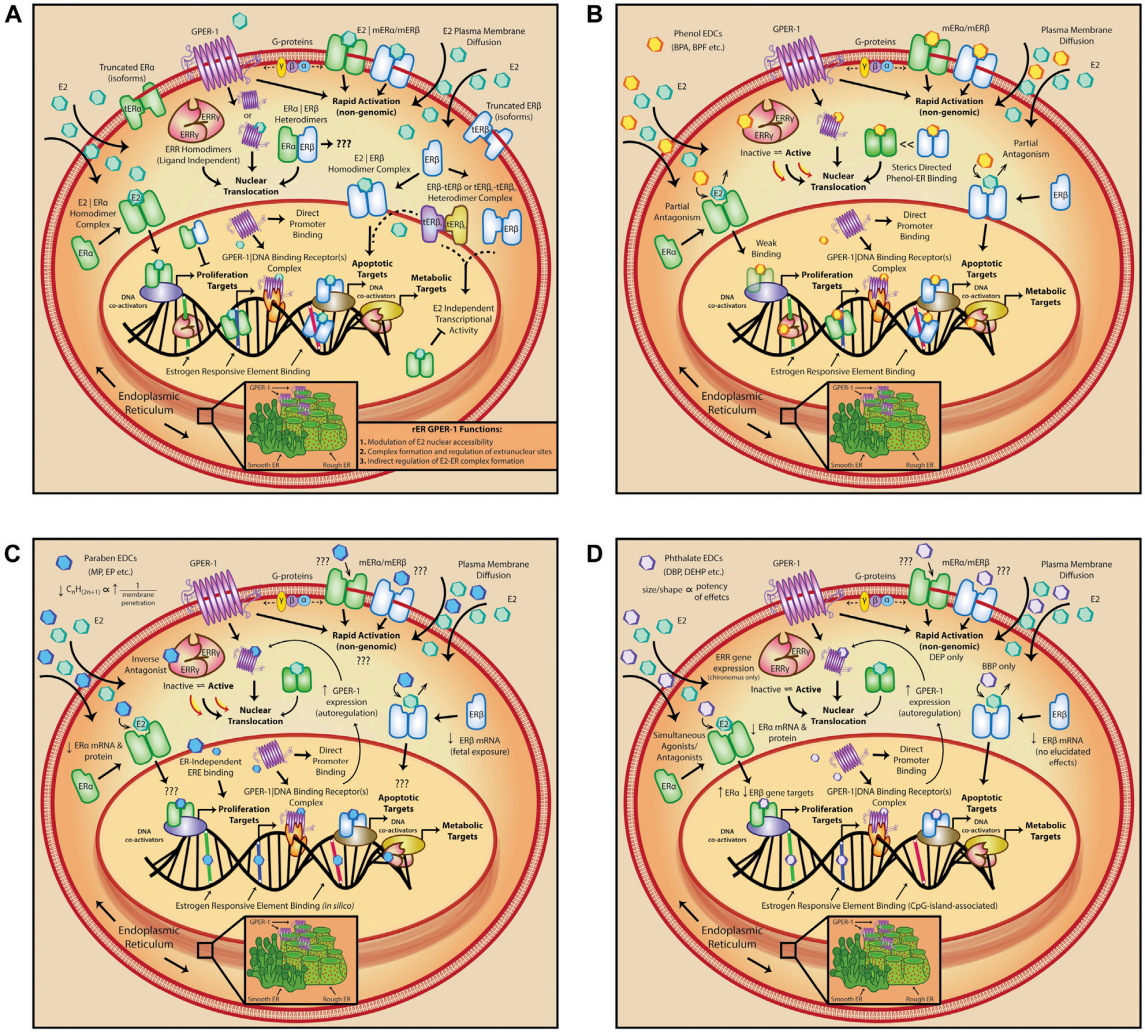
Estrogen signaling and interference by varying categories of EDCs. **(A)** Diversity of estrogen signaling modalities; estradiol (E2) activates several receptor targets to transduce both genomic and non-genomic signaling pathways. **(B)** Phenol-induced deregulation impacts both estrogen receptors, (ER)α and ERβ by genomic/intracellular partial antagonism; binding of phenols is also sterically directed. **(C)** Paraben EDCs exhibit similar deregulation of estrogen signalling pathways, including decreased expression of both estrogen receptors, (ER)α and ERβ, but their activity is largely limited on the basis of alkyl-group size. **(D)** Phthalate compounds display simultaneous agonistic and antagonistic effects on estrogen signaling modalities; similar to parabens, phthalate effects are also size and shape dependent. E2, estradiol; ERR, estrogen related receptor; tER, truncated estrogen receptor; GPER-1, G-protein coupled estrogen receptor 1; rER, rough endoplasmic reticulum; mER, membrane estrogen receptor; G-proteins, G-protein coupled receptor proteins; BPA, bisphenol A; BPF, bisphenol F; MP, methyl paraben; EP, ethyl paraben; BBP, benzyl butyl phthalate; DEP, diethyl phthalate; DEHP, di(2-ethylhexyl) phthalate.

Estrogens are critical for sexual and reproductive development especially in females and come in several similar chemical forms. Estradiol (E2) is the most abundant in cycling females, while estrone (E1) predominates in the postmenopausal period, and estriol (E3) and estrane (E4) predominate during pregnancy. Activity of endogenous estrogens, predominantly estradiol (E2) is primarily potentiated by the estrogen receptor-α (ERα) and ERβ, although an E2-specific G-protein coupled estrogen receptor-1 (GPER-1) has also been described. Hormone diffusion through the plasma membrane followed by receptor binding in the cytoplasm initiates canonical estrogen-to-ER signaling. Ligand-induced receptor dimerization and nuclear translocation lead to receptor-complexes that either directly bind estrogen responsive DNA elements (EREs) or complex with co-activators and co-repressors to regulate transcription of target genes (Levin and Hammes, 2016; Fuentes and Silveyra, 2019). ERα and ERβ can also heterodimerize, however their function is unclear, especially in contrast with the role of homodimers, such as proliferative ERα-ERα and suppressive/apoptotic ERβ-ERβ (Cowley et al., 1997; Papoutsi et al., 2009; Thomas and Gustafsson, 2011). ER signaling is complex and further diversified by the ability of receptors to localize at the plasma membrane in either truncated or full-length form, where they potentiate rapid non-genomic (non-translational) estrogen signaling (Figure 1) (Moss et al., 1997; Flouriot et al., 1998; Chambliss et al., 2000; Govind and Thampan, 2003; Hammes and Levin, 2007; Zhang et al., 2011). Other binding events at the cell membrane may also occur, such as interaction of full length ERα with G-proteins or with truncated isoforms of either ERα or ERβ (Kumar et al., 2007). Isoforms of ERβ, in particular, may further dimerize. Both homo- and heterodimers of ERβ isoforms have been characterized, each with its own distinct signaling modality. For example, homodimers of ERβ isoforms initiate estrogen-independent transcription whereas only heterodimers can initiate ligand-dependent transcription (Moore et al., 1998; Leung et al., 2006). Heterodimers of ERβ isoforms with full length ERβ can also form, and this increases the estrogen-independent transcriptional activity by four-fold. Activity of some, but not all, heterodimers of ERβ isoforms can be inhibited by ERα (Poola et al., 2005). Evidently, the complexity of estrogen signaling, and the diversity of ER modalities are critical for properly evaluating the effects of EDCs on individual immune populations. Although E2 generally has a higher affinity for its receptors compared to most EDCs, xenoestrogens can activate alternatively available ERs when estrogen is otherwise engaged. These EDC complexes may then act in retrograde upon complexed E2-ERs, ER-genes, or EREs to disrupt their activity.

#### Non-estrogen Receptor Targets

Alternative targets to the ERs have been extensively described. Estrogen-related receptors (ERRs), notably ERRγ, constitutively (sans-ligand) bind either estrogen-related and EREs or naked DNA and non-E2-related co-activators (Horard and Vanacker, 2003; Audet-Walsh and Giguere, 2015). GPER-1, a G-protein coupled receptor (GPCR) with preferential specificity for 17-β estradiol (E2β), potentiates the rapid, but transient, modulation of several signaling pathways (Filardo et al., 2000; Revankar et al., 2005; Thomas et al., 2005; Ge et al., 2013). Distribution of GPER-1 and its functionality have been comprehensively reviewed elsewhere (Cheng et al., 2011; Xu et al., 2019). In line with its structural classification as a seven transmembrane GPCR (7TMR), GPER-1 undergoes endocytosis, often in the absence of ligand, and has predominantly been localized in the perinuclear space. Constitutive internalization and localization of GPER-1 indicate its potential for nuclear activity, which is corroborated by identification of a putative nuclear localization sequence. This proposes that GPER-1 can initiate gene expression both by interaction with other DNA-binding nuclear receptors, or by direct E2 mediated promoter binding (Madeo and Maggiolini, 2010; Pupo et al., 2013; Rudelius et al., 2015). The way by which EDCs interfere with normal GPER-1 signaling is not well defined. Many EDCs can diffuse through the cell membrane and localize within the same cytoplasmic and/or perinuclear space as occupied by GPER-1. In this vicinity, EDCs can perturb local concentration dynamics or act directly on the GPCR binding site, and thereby interfere with GPER-1 target activation.

#### Estrogen and Estrogen-Related EDC Interactions

Competitive binding of EDCs to estrogen receptor targets has preceded interference on cell metabolism, nuclear receptor turnover, and hormone sensitization. As a result of binding to ERs, transcriptional enhancement along with modulation of other signaling pathways have been increasingly reported. The diversity of EDC categories also lends diversity to EDC effects. For example, certain xenoestrogens may disrupt activation of nuclear pathways by inherent ability to diffuse and translocate through the lipid membrane, where they promote rapid and direct gene expression. Other EDCs act more distally, on G-protein coupled receptors, to disrupt homeostatic expression of transcription factors, and latently affect gene expression (Yoon et al., 2014). Understanding the interplay of individual exposure, estrogen activity and immunomodulation, along with how this contributes to the collective phenomenon of autoimmunity, will lead to improved healthcare guidelines, reduced exposure burdens and novel therapeutics for the treatment of immune related disease.

### Phenols

BPA is the most extensively characterized phenol EDC and has been broadly used in numerous household products (Vandenberg et al., 2009). Past estimates approximated that 93% of Americans had detectable BPA in urine (0.4–149 μg/L) (Calafat et al., 2008). Despite regulatory reassurance in the past decade that BPA exposure is not deleterious within current limits, concern over adverse health effects eventually led to its tapered substitution in many consumer products, by either BPS or BPF. Limited assessment of these bisphenol analogs has permitted their unvalidated substitution in products under the label of safer or “BPA-free” alternatives. Emerging evidence, however, suggests not only that BPS has similar effects to BPA, but that it may, in fact, be more potent than its predecessor (Ferguson et al., 2019; Moon, 2019). Other phenols, such as alkylphenols, metabolites of ethoxylates, including NP monoethoxylates (NP1EOs) and diethoxylates (NP2EOs), all show evidence of bioaccumulation and are toxic (La Guardia et al., 2001). Not surprisingly, adverse phenol effects resulting in endocrine dysfunction have been extensively reported, including abnormal mammary gland development, decreased gonad and epididymis weights, and an increased risk of type 2 diabetes mellitus (Cha et al., 2017; Chamard-Jovenin et al., 2017; Yu et al., 2018). In the context of immunity, phenol interference on endocrine signaling and their activity as xenoestrogens hallmarks the critical influence of environmental factors on immune disease and emphasizes their function as exogenous immunomodulators.

#### Binding and Mode of Action

The ability of phenols to bind endogenous estrogen receptors, compete with endogenous ligand, and initiate activation of downstream pathways is central to understanding how they may modulate immune cells (Figure 1). In contrast with studies on non-genomic ER activation, phenols have been more extensively characterized in the context of genomic signaling. Relative to E2, BPA has reduced affinity for ERα (60–70% transactivation of E2) and exhibits preferential binding for ERβ (80%) over ERα. In consequence, phenols behave as partial antagonists in the presence of E2, in that binding of the EDC is less efficacious. Related factors such as bioaccumulation and tissue distribution of phenols, as well as generation of metabolites, render these EDC compounds with a complexity that exceeds E2 binding affinity alone (Matthews et al., 2001; Takayanagi et al., 2006). Moreover, variations in steric complexity seem to direct binding orientation of different phenols, which controls whether transduced effects will be agonistic (BPA) or antagonistic (BPC). This is also true of co-activator recruitment and/or binding, whereby BPA is a weak agonist, but bisphenol C (BPC) serves as an antagonist, and binding of both is typically favored in the presence of high co-activator concentrations (Delfosse et al., 2012). Despite this, bisphenols do act as full ERα agonists on cell growth, suggesting that this may be induced by direct ER-bisphenol binding to ERE DNA regions. Similar to ER-binding, phenol-GPER-1 agonist binding has been demonstrated, with relatively high affinity reported for BPA and NP (Thomas and Dong, 2006). In this context, GPER-1 on the rough endoplasmic reticulum can prevent phenol nuclear accessibility and allow E2-ER nuclear complex formation. Alternatively, pre-existing GPER-1 occupancy by E2, such as in the endoplasmic reticulum, can free up cytosolic or nuclear receptor targets for EDC binding and vice-versa. Thus, the physiological state of the organism at the time of exposure is critical for transduction of xenoestrogen effects and may help explain how EDCs function as triggers for disease in some individuals, but not in others. As well, synergistic effects of multiple pathways should similarly be considered. Phenols can further act as inverse antagonists on other receptor targets. BPA, in particular, has been shown to increase ERRγ constitutive activity, and could thereby lead to a deregulated cell metabolism with implications for both cancer and autoreactivity (Liu et al., 2007). Subsequent to their direct binding activity, phenols can augment the rate of receptor turnover. BPA decreases ubiquitination and degradation of ERβ, as well as intracellular ERα protein levels and mRNA transcripts (Masuyama and Hiramatsu, 2004; La Rosa et al., 2014). Similarly, BPF increases ERβ, however, other EDCs, such as NP, have yielded more complex, potentially sex and species specific effects upon receptor turnover (Sakimura et al., 2002; Seo et al., 2006; Okazaki et al., 2017). Collective observations have thereby surmised that many phenols display characteristics of selective ER modulators (SERMs), most notably in their ability to behave in a discriminatory manner (Delfosse et al., 2012). This functional classification is an important consideration for study design and should be deliberated when inferring results to human health.

The turnover rate of ERs and endogenous ligand are precisely balanced to control the downstream signaling necessary for proper function of immune cells. Docking of estrogen to ERs does not produce simple, all-or-nothing target activation. Instead, this binding event appears to induce a state that depends on the equilibrium between receptor and ligand. EDC-induced changes to such a system may impact either the perceived ligand concentration or the receptor availability, leading to complex and sometimes dichotomous results on immune function. Interpretation of immunological findings is further complicated by contrasting pre-clinical and clinical results, likely both influenced by study design and experiment dose.

#### Innate Immunity Effects

Dichotomy of immune findings is best illustrated by pre-clinical, innate immune findings. For example, phenols decrease downstream signaling and key regulators of inflammation, such as TNF-α, nitric oxide (NO), and NF-κB in both murine macrophages (BPA 228–22,800 μg/L) and immortalized macrophages (BPA 5,700–22,800 μg/L; NP 2,200 μg/L; OP 2,000 μg/L) (Byun et al., 2005; Yoshitake et al., 2008). Alternatively, BPA at similar doses can, instead, increase levels of NO, reactive oxygen species (ROS), NF-κB activation and mRNA transcripts of IL-1β and IL-10 pro-inflammatory cytokines. Interestingly, macrophage bactericidal function was improved following low-dose BPA exposure (0.10–10 μg/L), whereas administration of high dose BPA (100–10,000 μg/L) induced apoptosis, presumably in an oxidative stress-dependent manner (Yang et al., 2015). Dose-independent effects on cytokine secretion have been further observed for both BPA (0.10–1,000 μg/L) and NP (0.10–100 μg/L), such as increased transcripts for IL-1β, IL-10, TNF-α, IFN-γ, and MyD88. Consistently, NO metabolism was affected only by the high dose of BPA or NP (Xu et al., 2013). This dichotomy of results is not only biphasic but may reflect a species specificity for estrogen regulation that results in varying outcomes on inflammatory factors (Table 1).

**TABLE 1:** Diversity of immune effects by phenol EDCs.

	Dose (μg/L)	Species	Cell type	Immune impact	
*In vitro*					
BPA	100–10,000 (Yang et al., 2015)	Carp	MΦ	↑	NO, ROS, NF-κB, IL-1β mRNA and apoptosis
	0.10–10 (Yang et al., 2015)			↓	Bactericidal function
	228–22,800 (Byun et al., 2005) 5,700–22,800 (Yoshitake et al., 2008)		MΦ	↓	TNF-α, NO and NF-κB
	228–22,800 (Sakazaki et al., 2002)		N/A	↓	Lymphoproliferation (B cells > T cells)
	228–22,800^a^ (Jang et al., 2020)		Splenocytes	↓	Proliferation
	228 (Yurino et al., 2004)	Mouse	B1 B cells	↑	IgM
	1,000 (Bodin et al., 2015)		Splenocytes	↓	IL-10, TNF-α, IFN-γ and IL-4
				↑	GM-CSF
				↑	IFN-γ
				↑	MIP-1α
				↑	MIP-1β
	30, 300 μg/kg (Xu et al., 2019)		Serum (F)	↑	Development of T1D
	2,850–45,700 (Gostner et al., 2015)	Human	PBMCs (MΦ |T cells)	↓	Neopterin and IFN-γ
	0.02–22.8 (Ben-Jonathan et al., 2009) 0.228 (Valentino et al., 2013)^,72^		Adipocytes	↑	IL-6, TNF-α and IFN-γ
				↓	Adiponectin and glucose metabolism
				↑	JNK, STAT3 and NF-kB
	228–22,800 (Balistrieri et al., 2018)		PMNs	↑	ROS
				↓	Chemotaxis and bactericidal function
	22,800^a^ (Jang et al., 2020)		WiL2-NS (B cells)	↓	Proliferation
				↑	Sub G0/G1phase
				↓	G2/M phase and S phase
				↑	ROS
	228,000 (Alhomaidan et al., 2019)		Whole blood (DNA-HC)	↑	Hypochromicity/Stability
			Serum (SLE)	↑	Ab affinity/Recognition for BPA-DNA
BPF	20,000^a^ (Jang et al., 2020)	Mouse	Splenocytes	↓	Proliferation
	20,000^a^ (Jang et al., 2020)	Human	WiL2-NS (B cells)	↓	Proliferation
				↑	Sub G0/G1 phase, G2/M phase
				↑	ROS
BPS	7,500–25,000 ^a^ (Jang et al., 2020)	Mouse	Splenocytes	↓	Proliferation
	25,000^a^ (Jang et al., 2020)	Human	WiL2-NS (B cells)	↓	Proliferation
				↑	Sub G0/G1, G2/M phase and S phase
				↑	ROS
	2,200 (Yoshitake et al., 2008)	Mouse	MΦ	↓	TNF-α, NO and NF-κB
NP	220–22,000 (Sakazaki et al., 2002)		N/A	↓	Lymphoproliferation
	220 (Yurino et al., 2004)		B1 B cells	↑	IgM
OP	2,000 (Yoshitake et al., 2008)	Mouse	MΦ	↓	TNF-α, NO and NF-κB
*In vivo*					
BPA	100–1,000 (Xu et al., 2013)	Zebrafish	Embryo	↑	NO, NOS and ROS
	0.10–1,000^a^ (Xu et al., 2013)			↑	mRNA: IL-1β, IL-10, IFN-γ, MyD88 and TNF-α^a^
	300–3,000 μg/kg (*in-utero*) (Yoshino et al., 2004)	Mouse	Serum	↑	IL-4, IFN-γ and HEL-IgGs
				↑	Lymphoproliferation (CD8 > CD4)
	2.28–228 (Luo et al., 2016)		Splenic cells/Serum	↑	TH17 differentiation, IL-17, IL-21, IL-6 and IL-23 (F)
	300–350 mg/kg (Yurino et al., 2004)	Mouse (SLE)	B1 cells	↑	Anti-Br-RBC autoantibodies
	10,000 (Krementsov et al., 2013)	Mouse (EAE)	N/A	↑	First episode severity (F)
				↑	Relapse incidence (M)
	10 mg/kg (Brinkmeyer-Langford et al., 2014)	Mouse (TVID)	N/A	↑	Temporal onset of MS, disease severity
	50 μg/kg (Pirozzi et al., 2020)	Mouse	Serum/Liver	↑	mRNA: IL-6, TNF-α, IFN-γ, MyD88 and NF-κB
				↑	Progression of liver fibrosis
	1,000 (Bodin et al., 2015)		N/A	↑	Diabetic incidence
			Pancreatic islet cells	↑	Severity of insulitis
				+	Regulator of apoptosis
			MΦ	↓	Infiltrates and phagocytic function
	1,000–10,000 (Bodin et al., 2014)		N/A	↑	Diabetic incidence^a^ (F)
			Pancreatic islet cells	↑	Severity of insulitis^a^(F)
			MΦ	↑	Apoptosis
				↓	Infiltrates
NP	10–100 (Xu et al., 2013)	Zebrafish	Embryo	↑	NO, NOS and ROS
	0.10–100^a^ (Xu et al., 2013)			↑	mRNA: IL-1β, IL-10, IFN-γ, MyD88 and TNF-α^a^

Dose-dependent results are underlined. ^a^Immune effect at high dose(s) only; (F), female bias; (M), male bias; BPA, bisphenol A; BPF, bisphenol F; BPS, bisphenol S; NP, nonylphenol; OP, 4-tertiary-octylphenol; TLR-4, toll-like-receptor-4; NO, nitric oxide; ROS, reactive oxygen species.

Paradoxical results from clinical studies have likewise been reported. Human macrophages and T lymphocytes responding to BPA (2,850–45,700 μg/L) showed a dose-dependent decrease in neopterin and IFN-γ, respectively (Gostner et al., 2015). In contrast, production of IL-6, TNF-α and IFN-γ from human adipocytes was increased after exposure to BPA (0.02–22.8 μg/L Ben-Jonathan et al., 2009; 0.228 μg/L Valentino et al., 2013). This corresponded with a lowered production of the anti-inflammatory hormone, adiponectin, and an impaired glucose metabolism (Ben-Jonathan et al., 2009; Valentino et al., 2013). In the context of clinical obesity and chronic inflammation, low-dose EDC exposure effects suggest that EDCs may exacerbate or even cause autoimmunity, including post-infection autoreactivity of metabolic etiology. Such potential has been identified for BPA, which increased activation of JNK, STAT3 and NF-kB signaling pathways. Conversely, adipogenesis was unaffected despite previous pre-clinical reported effects (BPA 0.228 μg/L; Valentino, D'Esposito et al., 2013; Ariemma, D'Esposito et al., 2016).

In other clinical findings, phenol effects were transduced by GPER-1 activation to induce human polymorphonuclear neutrophils (PMNs) toward a pro-inflammatory phenotype (Rodenas, Tamassia et al., 2017). Despite limited research into EDC effects upon PMNs, administration of BPA (228–22,800 μg/L) did increase ROS production in these cells, in a dose-dependent manner. ROS activity was also associated with diminished bactericidal function and inhibition of PMN chemotaxis, whereas phagocytic function remained unaffected (Balistrieri, Hobohm et al., 2018).

#### Adaptive Immunity Effects

Phenols have additionally been characterized for their effects on adaptive immunity. Gestational BPA treatment (300–3,000 μg/kg) alters the humoral response to immunization, leading to higher levels of antigen specific immunoglobulin (Ig) Gs and increased lymphoproliferation (Yoshino et al., 2004). Increased IFN-γ and IL-4 were also noted as a result of BPA effects on T cell helper phenotypes, particularly TH1 differentiation. Furthermore, despite an overall increase in T cells, the ratio of CD8/CD4 T cells was skewed in favor of CD8 T cells. Collectively, these results suggest that phenols may prime the humoral response toward sustained inflammation (Yoshino et al., 2004). In the context of pathogen response or autoimmunity, sustained humoral activation as induced by BPA can degenerate into chronic inflammation and the onset or exacerbation of autoreactivity. This is further substantiated by evidence of BPA-induced (2.28–228 μg/L) increased differentiation of TH17 cells, including increased secretion of IL-17 and IL-21, as well as TH17 differentiating cytokines, IL-6 and IL-23. Such changes were altogether dose-dependent and sex-specific (female bias), further suggesting that BPA alters dynamics of adaptive immunity and leads toward a proinflammatory phenotype that may cause adverse health outcomes (Luo, Li et al., 2016).

In contrast, *in vitro* treatment with BPA (2,280–22,800 μg/L) or NP (220–22,000 μg/L) decreased lymphoproliferation, with BPA demonstrating preferential inhibition of B cells over T cells (Sakazaki et al., 2002). Comparable with BPA (22,800 μg/L), analog compounds, BPF (20,000 μg/L) and BPS (7,500 and 25,000 μg/L), decreased splenocyte proliferation and augmented human B cell cycle transitions. The reduced viability of human B cells, likely as a result of enhanced ROS production by all three bisphenols, was comparable with effects previously described for innate cells (Yang et al., 2015; Jang et al., 2020). This ability of EDCs to downregulate B cell responses may be critical in the context of viral or other chronic infections, which have been linked to the onset of several autoimmune phenotypes (Box 1). By affecting TH differentiation, altering the distribution of CD8 to CD4 T cells, and diminishing B cell responses, EDCs may directly contribute toward an ineffective adaptive response, one which may allow pathogen escape and influence the development of chronic inflammation and self-reactivity.

Box 1 **Relevance of EDC-immune modulation to COVID-19 disease and post-infection autoimmunity.**

Modulation of adaptive immune responses by EDCs presents critical considerations for the current COVID-19 pandemic, caused by the SARS-CoV-2 virus. Recent studies have linked the incidence and severity of SARS-CoV-2 infection with development of post-infection autoimmunity, including diabetes, Guillain-Barre syndrome and autoimmune hemolytic anemia (Dalakas, 2020; Lazarian et al., 2020; Mallapaty, 2020). Herein, we describe findings that identify immune modulatory activity as similarly reported for COVID-19 disease but following EDC exposure. In both contexts, there exists an increased predisposition for autoreactivity. Therefore, we underscore the likelihood of synergism between EDC-induced immune dysfunction and SARS-CoV-2 infection. The sex-bias of immune self-reactivity, conveyed by estrogen mimicry with EDCs, may also explain the increased severity of COVID-19 in males as well as the age-dependency of infection frequency (F: 10–50 years, estrogen^high^; M: 10< and >50 years, testosterone^low^) (Scully et al., 2020). To illustrate, adaptive immunity in females may be better equipped to initiate antiviral defenses in the initial stages of infection, in part likely due to the same processes responsible for the female autoimmune bias. Several of the studies we outlined demonstrate the capacity of EDCs to increase the expression of cytokines including IL-6, IL-1β, TNF-α, and IFN-β. Therefore, it is plausible that under noninfectious circumstances, elevated levels of these cytokines may result in an increased baseline state of immune activation. During a pathogen defense response, this heightened state may convey an advantage. However, without a specific target, it could trigger autoreactivity. In contrast, male sex-hormones (testosterone^high^/estrogen^low^), which are otherwise preventive against EDC exposure effects and autoimmunity, could dampen the initial response to SARS-CoV-2 infection. This would result in increased viral burden, which may thereby trigger hyperinflammatory syndrome and severe COVID-19 disease in males. Interestingly, early data has suggested an association between male-sex and viral burden duration (Scully et al., 2020). Furthermore, the risk to develop severe disease and mortality rate were both found to be male-biased (Maleki Dana et al., 2020; Scully et al., 2020)

#### Autoimmunity

Exposure to phenols is a potential risk factor for autoimmunity, however studies are similarly limited by complex, often biphasic results. In animal models of autoimmune disease, such as systemic lupus erythematosus (SLE), long-term BPA exposure (300–350 mg/kg) enhanced production of anti-Br-RBC autoantibodies. Elevated levels of IgM secretion from B1 cells have also been reported following exposure to both BPA (228 μg/L) and NP (220 μg/L) (Yurino et al., 2004). Gestational BPA (10,000 μg/L) followed by induction of autoimmune encephalomyelitis (mild EAE; MOG_35–55_/CFA-only) did not affect the clinical course of disease. Conversely, trends in severity of EAE were observed, such as an increased disease score during the first EAE episode in female mice and a higher relapse incidence in male mice (Krementsov et al., 2013). Similarly, an earlier onset of MS symptoms and worsened disease severity were shown in a virus-induced demyelination model of MS (BPA 10 μg/kg) (Brinkmeyer-Langford et al., 2014). Interestingly, colitis was more severe in MS mice exposed to BPA, which corroborates clinical data on the co-occurrence of MS and inflammatory bowel diseases (IBDs) in humans (Kimura et al., 2000; Alkhawajah et al., 2013; Brinkmeyer-Langford et al., 2014).

Consideration of phenols in the context of other autoimmune conditions provides additional evidence for their harmful effects. For example, EDC levels (BPA 1,000 μg/L) relevant to human exposure increased diabetic incidence and corresponded with higher grade insulitis in pancreatic islets, concurrent with decreased macrophage infiltrates of lowered phagocytic potential (Bodin et al., 2015). Similar findings on type-1 diabetes identify BPA as pro-apoptotic for pancreatic islet cells, which suggests a mechanism by which BPA promotes self-antigen auto-activation and diabetic onset (Bodin et al., 2014; Bodin et al., 2015). Furthermore, high-fat diet and BPA exposure (50 ug/kg) were associated with immune-metabolic dysfunction, characterized by increased transcripts for toll-like-receptor-4 and related signaling pathways, such as NF-kB and NLRP3 inflammasome, which amplified proinflammatory cytokine production to promote liver damage (Pirozzi et al., 2020). More specifically, in diabetic animals, decreased levels of secreted IL-10, TNF-α, IFN-γ and IL-4 have also been reported. Animal findings thus suggest that BPA and other phenols can alter the severity of autoimmune disease, likely through a mélange of effects, including increased production of autoantibodies, altered cytokine secretion and decreased macrophage scavenging. Altogether, these changes contribute to the persistence of self-antigen and may exacerbate autoimmunity (Bodin et al., 2015). There is also evidence that phenol exposure could contribute to sex-bias in autoimmune disease outcomes. In non-obese diabetic mice, BPA affected both development and pathology of T1D in a sex-specific manner. In females, development of T1D was accelerated and accompanied by a shift in proinflammatory immune factors, whereas disease development in males was delayed, associating with an increase in anti-inflammatory factors (Xu et al., 2019). These results highlight the potential of phenol EDCs to induce sex-specific changes in the development and course of autoimmune diseases.

In humans, the role of phenol EDCs in autoimmunity is less defined. Nevertheless, clinical studies have provided compelling evidence on the detriment of EDCs to human autoimmunity (Table 2). When isolated DNA from healthy individuals was exposed to BPA (228,000 μg/L), formation of DNA-BPA complexes and BPA-induced conformational changes (48.2% hypochromicity, 260 nm) were observed. Relative to native DNA, DNA-BPA complexes had increased stability, which suggests a novel process for how environmental factors may influence the development of self-reactive autoantibodies. In conformity, serum IgG antibodies from SLE patients showed affinity and recognition for such DNA-BPA complexes, but were otherwise undetected by IgG antibodies purified from healthy individuals, presumably because the latter have no affinity for self-antigens, such as anti-double stranded DNA (dsDNA) recognition. Interestingly, the SLE IgG antibodies showed preferential recognition of BPA-altered DNA over native DNA, which could help explain how an otherwise weak immunogen (dsDNA) is enhanced to autoimmunity (Rekvig and Mortensen, 2012; Alhomaidan et al., 2019). An impact of phenols has also been reported in autoimmune thyroid disease, where serum BPA showed a positive trend with antibody levels for thyroglobulin (TgAb), thyroid peroxidase (TPOAb) and thyroid receptor (TRAb). BPA was also an independent predictor of TgAb and TPOAb, but not TRAb (Chailurkit et al., 2016). Whether modulation of immune populations by phenols has the capacity to influence the onset or progression of autoimmune disease remains controversial, however, animal findings implicate immune effects that are dependent on several factors including study design (Table 1).

**TABLE 2:** EDC population effects.

EDC compound	Population/sample	Biomarker and/or disease association	
*Phenols*			
BPA	Thyroid autoimmunity/serum (Chailurkit et al., 2016)	↑	TgAb
		↑	TPOAb
		↑	TRAb
*Parabens*			
MP	Case report (Henry et al., 1979; Macy et al., 2002)	↑	Hypersensitivity and contact urticaria
	Urine [pregnant (F)] (Watkins et al., 2015)	↓	C-reactive protein
		↑	IL-6
		↑	IL-10
		↑	IL-1β
	Urine (M) (Quiros-Alcala et al., 2019)	↑	Asthma morbidity
EP	Urine (Liu et al., 2019)	↑	Gestational diabetes mellitus
PP	Urine [Pregnant (F)] (Watkins et al., 2015)	↑	Oxidative stress
	Urine^a^ (Ward et al., 2020)	↓	Diabetic incidence
	Urine (M) (Quiros-Alcala et al., 2019)	↑	Asthma morbidity
BP	Urine/Serum [pregnant (F)] (Watkins et al., 2015)	↑	Oxidative stress
		↑	IL-1β
		↑	IL-6
		↑	IL-10
		↑	TNF-α
	Urine^a^ (Ward, Casagrande et al., 2020)	↓	Diabetic incidence
*Phthalates*			
MEP	Human cord blood (Herberth et al., 2017)	↓	T Regulatory cells
MiBP	Human cord blood (Herberth et al., 2017)	↓	T Regulatory cells
DEHP	Urine (Yang et al., 2019)	↑	Anti-HBs IgM responses following post-natal HBV immunization
		↑	Modulation of gut microbiota composition
DiBP	Urine (children) (Castro-Correia et al., 2018)	↑	New onset type 1 diabetes mellitus

^a^Immune effect at high detected concentrations; (M), male bias; BPA, bisphenol A; MP, methyl paraben; EP, ethyl paraben; PP, propyl paraben; BP, butyl paraben; MEP, monoethyl paraben; MiBP, monoisobutyl phthalate; DEHP, di (2-ethylhexyl) phthalate; DiBP, diisobutyl phthalate.

### Parabens

The paraben category of EDCs has also been described for its effects on immunity (Tables 2 and 3). The size of alkyl groups is a defining characteristic for paraben compounds and has been proposed to correlate directly with the degree of ERα and ERβ estrogenic activity (Gomez et al., 2005). However, the size of paraben alkyl groups is correlated with their penetration potential across the cell membrane. This suggests that although higher molecular weight parabens may have increased estrogen-like activity, exposure to such compounds from goods and other commodities is largely limited to their less estrogenic counterparts. In routes of direct exposure, such as intravenous administration, bioaccumulation is also minimal, with paraben levels quickly declining from the serum or maintained at nontoxic levels (Andersen, 2008). Nevertheless, paraben-induced chromosomal aberrations, including gaps, breaks, exchanges and rings have been described, suggesting that lower molecular weight compounds that cross the membrane may directly impact expression of immune genes, despite having lower estrogenic activity (Matsuoka et al., 1979). Alternatively, contributions of the endocrine system in normal immune function are vital, such that even milder disruptions by parabens may be detrimental to human health.

**TABLE 3:** Diversity of immune effects by paraben EDCs.

	Dose (μg/L)	Species	Cell type	Immune impact	
*In vitro*					
BP	2 (×10^8^) (Matsuoka et al., 2018) (Indirect/sensitization)	Mouse	Brachial LN cells	↑	IL-4 and IFN-γ
			Dendritic cells	↑	Skin to dLN trafficking
	1.17 (×10^–2^) (Bairati et al., 1994)	Human	Lymphocytes	↓	Lysozyme release
HP-DP	2,360–2,780 (Uramaru et al., 2014)	Rat	Peritoneal mast cells	↑	Histamine release
PP-DDP	20,800–30,600^a^ (Uramaru et al., 2014)	Rat	Peritoneal mast cells	↑	Histamine release
IPP	20,800 (Uramaru et al., 2014)	Rat	Peritoneal mast cells	↑	Histamine release
*In vivo*					
BP	200 mg/kg (Hegazy et al., 2015)	Rat (M)	N/A (brain lysates)	↑	NO, IL-6 and TNF-α
				↓	IL-1β mRNA

#### Binding and Mode of Action

Paraben-ER activity and binding mechanism depends on the octanol-water partition coefficient (*K*_ow_), an index of cell membrane transfer. Parabens with a relative high *K*_ow_, such as BP, can diffuse across the cell membrane and directly bind DNA *in silico* (Manzetti, 2018). Ability to bind DNA directly is not exclusive to BP. Transcriptional and translational products of ERE-genes have also been reported following high dose (1,000 mg/kg/d) exposure to isopropyl and isobutyl parabens. The concurrent decrease in mRNA and protein expression of ERα further suggests that parabens may not always follow an ER-to-ERE DNA activation pattern, and can instead directly bind to the ERE (Vo and Jeung, 2009). Alternatively, PP induces morphogenic changes in glandular structures via activation of both ERα and ERβ and GPER-1 (Marchese and Silva, 2012). Exposure to MP (3 μg/L), PP (3.6 μg/L) or BP (3.9 μg/L), can also increase GPER-1 gene and protein expression, which indicates that parabens can activate a GPER-1 autoregulatory feedback loop (Wrobel and Gregoraszczuk, 2015). Additional activity of parabens on other estrogen targets involves the inverse antagonism of ERRγ. Parabens (methyl-to-benzyl parabens) are recruited *in silico* to the ERRγ active site, and form bonds with active site residues (Zhang et al., 2013). Binding of parabens to ERα or ERβ conformations that transduce non-genomic signaling has not yet been described, likely due to the size limitation on membrane permeability. However, in the intercellular space, size-excluded parabens could preferentially target surface ERα or ERβ to activate non-genomic signaling. Alternatively, the alkyl bulk of parabens may impede ligation to the active site of surface ERs. Nevertheless, activation of estrogen targets by parabens with a high K_ow_ offers sufficient insight to raise concern regarding the interference of paraben EDCs with normal endocrine signaling in immune populations.

#### Innate Immunity Effects

Effects of paraben exposure on innate immunity are often derived from cosmetic allergology studies assessing the safety of personal care products. This restricts findings within a narrow niche of innate immune cells, specifically those that relate to allergic sensitivities. Nevertheless, findings can help inform on effective dosages and exposure schedules. Notably, dose ranges often fall within or surpass the doses at which activation of ERs has been described. For example, straight-chain parabens spanning from heptyl-to-decyl (low dose HP-DP, 2,360–2,780 μg/L) or pentyl-to-dodecyl (high dose PP-DDP, 20,800–30,600 μg/L) cause a significant release of histamine from degranulating mast cells (Uramaru et al., 2014). This is also sustained by exposure to the branched isopentyl paraben (IPP, 20,800 μg/L). MP causes immediate hypersensitivity, including contact urticaria, although it is unclear whether paraben-reactivity is a result of innate or adaptive activation, the latter of which would be an anti-paraben allergic recall (Henry et al., 1979; Macy et al., 2002). To date, discerning these effects has been limited by unsuccessful detection of anti-paraben IgE antibodies in the serum. However, this does not rule out a paraben-induced adaptive recall. IgE antibodies have a relative short-half life, which temporally limits their detection, and may lead to the incorrect assumption that paraben hypersensitivity is a localized innate response. Alternatively, other immunoglobulin classes, such as IgG antibodies, may be involved in activation of innate cells, transducing effects otherwise perceived as immediate reactivity to parabens (Kokubu et al., 1989). A more detailed discussion is included in the adaptive immunity subsection on paraben effects.

Observations independent of allergic innate responses have been modestly reported. In the brain, BP (200 mg/kg/d) increases the levels of NO, IL-6 and TNF-α but downregulates IL-1β (Hegazy et al., 2015). Peripherally, it can also enhance dendritic cell (DC) trafficking to draining lymph nodes (dLN) and increases the secretion of IL-4 and IFN-γ from dLNs (Matsuoka et al., 2018). During pregnancy, exposure to BP and PP associate with higher measures of oxidative stress, lower expression of c-reactive protein, and changes in cytokine secretion. BP for example, increases IL-1β, IL-6, IL-10, and TNF-α levels, whereas MP increases only IL-6 and IL-10 and decreases levels of IL-1β (Watkins et al., 2015). The ability of parabens to influence cytokine dynamics implicates not only innate but also adaptive responses, as the latter depend upon the precise balance of inflammatory factors to mount the appropriate humoral defense.

#### Adaptive Immunity Effects

Effects of parabens on adaptive immunity have primarily remained unexplored, perhaps because of the restrictive interest in parabens outside of cosmetics and personal care products. Alternatively, parabens can be erroneously overlooked since they have minimal bioaccumulation potential and relatively lower toxicity compared to other EDC categories. Nevertheless, clinical case reports identify anomalous serology characterized by both anti-paraben antibodies and paraben-associated auto anti-Jk^a^ antibodies (Judd et al., 1982; Judd et al., 2001; Gniadek et al., 2018). Although characterization of paraben-specific IgE antibodies is limited by their half-life, sensitization with BP and HP followed by challenge produces a significant allergic reaction (Uramaru et al., 2014). This suggests that prior paraben priming is sufficient to generate recall upon antigen re-presentation and is a hallmark feature of adaptive immunity. Interestingly, patients presenting with intractable dermatitis resistant to typical corticosteroid creams, achieve rapid and complete recovery when administered paraben-free corticosteroid formulations. This indirectly confirms the existence of paraben-specific antibodies, although it does not offer an explanation as to how parabens may trigger an adaptive response (Guyton et al., 2005). One possibility involves a combination of effects that involves both innate and adaptive mechanisms. Parabens acting on innate cells result in an unbalanced inflammatory milieu that recruits adaptive immune cells. In turn, recruited cells activate in the presence of cytokines and are then primed by exogenous parabens, generating antibodies with paraben affinity. In the context of dermatitis, this presents an easily remediable effect of exposure. However, such immunomodulation may be detrimental when paraben exposure occurs simultaneous with an ongoing innate or adaptive response, and could trigger hyperinflammatory syndrome and autoreactivity, respectively.

#### Autoimmunity

The role of parabens in autoimmunity is similarly limited to a few exploratory studies. To the best of our knowledge, no findings have been reported for paraben effects on immune cells that drive autoimmunity, such as B cells or T cells. However, an association of EP urinary levels and gestational diabetes has been reported, which suggests an increased risk of diabetes in later life (Liu et al., 2019). In contrast, high concentrations of methyl-to-butyl parabens in urine were associated with lower odds of diabetes overall (Ward et al., 2020). This suggests that exposure to parabens in the context of diabetic autoimmunity is more complex and requires a molecular approach for validation.

In children, sex-biased associations have been determined between urinary concentrations of methyl and propylparaben, whereby boys have increased asthma morbidity, as modeled by emergency department visits. A similar male-bias has also been reported in paraben-related triclosan exposure and food sensitization among children, further suggesting that EDCs influence human immune disease outcomes in a sex-specific manner (Savage et al., 2012; Quiros-Alcala et al., 2019). Studies on central nervous autoreactivity have shown that paraben exposure, particularly BP (1.17 × 10^–2^ μg/L), inhibits lysosomal enzyme secretion, which is otherwise elevated extracellularly in MS patients (Bairati et al., 1994; Guyton et al., 2005). Nevertheless, combined findings illustrate the diversity of effects with which paraben EDCs influence immunity.

### Phthalates

Phthalates comprise the third category of EDCs. Although a variety of deleterious effects have been identified, phthalate compounds have commonly been described in reference to phthalate syndrome, a condition that causes developmental changes to the reproductive system. Similar to parabens, the strength of phthalate effects is conveyed by the shape (e.g., branching) and length of the ester side chains (Takeuchi et al., 2005). Compounds with relative high potency contain linear ester sidechains of 4–6 carbons, whereas shorter or longer phthalate esters tend to cause less severe effects (Council, 2008). Nevertheless, many of these compounds are known endocrine disruptors and have adverse outcomes, including multigenerational and transgenerational reproductive dysfunction (Table 2) (Kay et al., 2013; Zhou et al., 2017). The role of phthalates in reproductive health emphasizes their relevance for all aspects under endocrine control, including innate and adaptive immune responses.

#### Binding and Mode of Action

Like members of other EDC categories, phthalates both activate and disrupt endocrine signaling, particularly pathways regulated by estrogen and its receptors (Figure 1). Although non-genomic activation has been defined for diethyl phthalate (DEP), it has remained unexplored for other compounds (Kumar et al., 2014). Nuclear effects, however, are generally more common. For example, butyl benzyl phthalate (BBP; 3,120 μg/L) caused demethylation of ERα promoter-associated CpG islands and increased ERα gene expression (Kang and Lee, 2005). In GPER-1^+^ cells, BBP (31,200 μg/L), DBP (2,800 μg/L), and DEHP (3,900 μg/L) all led to increased proliferation. In the absence of GPER-1, this effect was undetected for all phthalates, which suggests that they initiate proliferation via GPER-1 (Kim et al., 2004). Additionally, both DBP and diisopentyl phthalate (DiPeP) (500 mg/kg/d) increase GPER-1 gene expression, whereas DiPeP at a lower dose (125–250 mg/kg/d) decreases ERα without affecting levels of ERβ (Curi et al., 2019). Interestingly, phthalates may act simultaneously as agonists and/or antagonists, primarily via ERα; only BBP has been shown to have activity on ERβ. Binding of phthalates to other estrogen targets, including ERRs, has not been extensively characterized. Only one study found that DEHP (50,000 μg/L) increased ERR gene expression (Park and Kwak, 2010). Nevertheless, collective data (Figure 1) suggests that phthalates can exert effects via both ERα and GPER-1 and have the potential to disrupt cells that depend on estrogen signaling, which includes cells of the immune system.

#### Innate Immunity Effects

Phthalate compounds and their metabolites have been identified as adverse modulators of innate immunity (Table 4). Perinatal DEHP (60 and 600 μg/kg/d) alters inflammatory functions of peritoneal macrophages and results in suppression of TNF-α and IL-1β gene expression. In contrast, gene expression for IL-6 was significantly upregulated, suggesting that phthalate exposure contributes to the dysregulated continual production of IL-6, which has been associated with the onset of various diseases. Interestingly, both DEHP and its metabolite, mono-(2-ethylhexyl) phthalate (MEHP, 20 μg/L), reduced macrophage phagocytosis (Li et al., 2018). Because phthalates are known to leak from plastic goods and accumulate as contaminants in soil and water sources, there is concern that metabolite exposure may lead to inadvertently persistent exposure. Similar cytokine dysregulation in human innate immune cells has also been reported. DEP (22,200 μg/L) and DBP (27,800 μg/L) upregulate secretion of IL-6, CXCL8 and IL-10 secretion and inhibit TNF-α secretion in macrophages (Hansen et al., 2015). DEHP (3,900 μg/L) further modulates the differentiation and maturation of PBMC-derived DCs (Ito et al., 2012). Even at low doses, both DEHP (39 μg/L) and BBP (31 μg/L) suppress expression of type I interferons, such as IFN-α and IFN-β, from plasmacytoid DCs (Kuo et al., 2013). Collectively, animal and human studies are congruent in implicating phthalates in cytokine dysfunction that can drive aberrant innate immune responses.

**TABLE 4:** Diversity of immune effects by phthalate EDCs.

	Dose (μg/L)	Species	Cell type	Immune impact	
*In vitro*					
MBP	22,200 (Hansen et al., 2015)	Human	T Cells	↑	IL-6
MEHP	20 (Li et al., 2018)	Mouse (F)	M Φ	↓	Phagocytosis
DEP	22,200 (Hansen et al., 2015)	Human	M Φ	↑	IL-6
				↑	CXCL8
				↑	IL-10
				↓	TNF-α
			T Cells	↓	IL-2
				↓	IL-4
				↓	TNF-α
				↓	IFN-γ
DBP	27,800 (Hansen et al., 2015)	Human	MΦ	↑	IL-6
				↑	CXCL8
				↑	IL-10
				↓	TNF-α
			T Cells	↓	IL-2
				↓	IL-4
				↓	TNF-α
				↓	IFN-γ
BBP	31^a^ (Kuo et al., 2013)	Human	Plasmacytoid dendritic cells	↓	IFN-α
			T Cells	↑	IFN-β
				↓	IFN-γ
				↑	IL-13
DEHP	≥1,560 (Martins et al., 2015)	Rainbow trout	B Cells	↓	Proliferation
	≥6,250 (Martins et al., 2015)		IgM plasmablasts/Plasma cells	↓	Proliferation
	60 and 600 μg/kg/d (Li et al., 2018)	Mouse	MΦ	↓	TNF-α mRNA (F)
				↓	IL-1 mRNA (F)
				↑	IL-6 mRNA (F)
	3,900 (Ito et al., 2012)		Dendritic cells	↑	Differentiation and maturity
	30, 300, 3,000 μg/kg (Han et al., 2019)		T_FH_	↑	Bcl-6
				↑	c-MAF
				↑	IL-21
				↑	IL-4
	39^a^ (Kuo et al., 2013)	Human	Plasmacytoid dendritic cells	↓	IFN-α
				↑	IFN-β
			T Cells	↓	IFN-γ
				↑	IL-13
*In vivo*					
BBP	3,000 (Elter et al., 2020)	Mouse	N/A	↑	Severity of RA in progeny
			Serum	↑	IgG1
				↑	IgG2a
			Splenocytes	↑	IFN-γ
				↑	IL-17
DEHP	60 and 600 μg/kg/d (Li et al., 2018)	Mouse	MΦ	↓	TNF-α gene expression
	60 μg/kg/d (Li et al., 2018)			↓	Phagocytosis
	30, 300, 3,000 μg/kg (Han et al., 2014)		T_FH_	↑	Co-stimulatory activity
				↑	SLAMF1 and SAP
				↑	Germinal center formation
	11.3–13.3 mg/kg/d (Hirai et al., 2015)	Mouse (M with EAO)	MΦ and other cells	↑	Number IFN-γ+
			Lymphocytes	↑	Infiltrates
			Serum	↑	Anti-testicular germ cell autoantibodies

^a^Immune effect at high dose(s) only; (F), female bias; (M), male bias; MBP, monobutyl phthalate; MEHP, mono-(2-ethylhexyl) phthalate; DEP, diethyl phthalate; DBP, dibutyl phthalate; BBP, butyl benzyl phthalate; DEHP, di (2-ethylhexyl) phthalate; TFH, follicular helper T cells.

#### Adaptive Immunity Effects

Phthalate-induced dysregulation of adaptive response has unsurprisingly resulted from both parent esters and their metabolites. DEP (22,200 μg/L) and DBP (27,800 μg/L) impaired activation-induced T cell secretion of IL-2, IL-4, TNF-α and IFN-γ, but not levels of IL-6 or IL-10. In contrast, the monoester metabolite of DBP, monobutyl phthalate (MBP, 22,200 μg/L) upregulated expression of IL-6, suggesting that phthalate compounds are twice as immunomodulatory (Hansen et al., 2015). In their intact form, phthalates may disrupt signaling pathways that control normal cytokine expression, such as E2-mediated transduction. However, once inside the cell, they can also be processed into various metabolites. This extends their activity and sometimes produces effects that are independent of the parent compound. Effects induced by monoester metabolites have been reported, including an associated reduction of cord blood *T*_reg_ cells by both monoethyl (MEP) and monoisobutyl phthalate (MiBP) (Herberth et al., 2017). Nevertheless, not all studies report metabolite activities but they do corroborate adaptive immune dysfunction. For example, DEHP (39 μg/L) and BBP (31 μg/L) suppress IFN-γ and can increase IL-13 secretion from CD4 T cells (Kuo et al., 2013). In B cells, DEHP inhibits proliferation (≥1,560 μg/L), which causes a significant reduction in IgM-secreting plasmablasts and plasma cells (≥6,250 μg/L) (Martins et al., 2015). In contrast, DEHP (30, 300, 3,000 μg/kg) can also increase expression of SLAMF1 and SAP in T_FH_ cells, thereby acting as an immunoadjuvant to promote co-stimulatory activity. DEHP also leads to enhanced formation of germinal centers by elevating expression of transcription factors, Bcl-6 and c-MAF in T_FH_ cells, as well as IL-21 and IL-4 cytokines (Han et al., 2014; Han et al., 2019). Interestingly, early-life DEHP exposure elevates secretion of virus-specific IgM following post-natal hepatitis B immunization. This, however, alters the gut microbiota in newborns and could have lasting repercussions on immune responses in later-life, including onset of autoimmune disease (Yang et al., 2019).

*Autoimmunity*. Contributions of phthalates to autoimmunity have been thoroughly characterized, especially their potential to exacerbate, or perhaps, induce autoreactivity. In testicular autoimmunity, DEHP (0.01% or 11.3–13.3 mg/kg/d) increases severity of mild experimental autoimmune orchitis (EAO) and is accompanied by an increase in IFN-γ^+^ cells and overall macrophages. Elevated lymphocytic infiltrates and anti-testicular germ cell autoantibodies further suggest that phthalates can exacerbate autoimmune disease (Hirai et al., 2015). In rheumatoid arthritis (RA), maternal BBP administration (3,000 μg/L) similarly increases the prevalence and severity of RA in the progeny, illustrating a transgenerational effect of phthalate exposure. This is associated with elevated levels of serum IgG1 and IgG2a, as well as enhanced secretion of IFN-γ and IL-17 from splenocytes (Elter et al., 2020). DEHP (7.5 mg/kg) can also increase levels of autoantibodies and associated proteinuria-induced renal dysfunction (Lim and Ghosh, 2005). Effects of phthalates on other autoimmune conditions have also been identified. In children with new onset of type 1 diabetes, higher levels of DiBP metabolites were detected by comparison with healthy controls (Castro-Correia et al., 2018). Furthermore, although the temporal context of immune deregulation is yet debated, inappropriate inflammatory responses and altered functions of lymphocytes have been proposed to be causative for and/or correlative with type 2 diabetes (de Candia et al., 2019). At levels relevant to human exposure, phthalates induce both transient and life-long metabolic dysfunction, including an increased risk of type 2 diabetes and insulin resistance (Radke et al., 2019). Combined data thus illustrates that phthalates directly and indirectly (transgenerationally) disrupt the normal function of both innate and adaptive immune processes, which may trigger onset of autoimmunity, especially in predisposed individuals. In addition, phthalate immunomodulation may lead to somewhat milder, but chronic, effects, such as persistent inflammation. This disruption in cytokines may, over time, provide a favorable context for self-reactivity to develop, even in individuals otherwise disinclined to autoimmune disease.

## Discussion

The sex-bias of disease susceptibility has remained an unresolved phenomenon of autoimmunity, including in MS, RA and SLE. This puzzling bias has also gained momentum with post-infection viral autoimmunity and has particular relevance to the current SARS-CoV-2 pandemic. In considering these two, seemingly unrelated, settings, we draw attention to the incidence rate of female-biased autoimmunity and emphasize its steady increase, which has suggested a role for environmental factors. Similar influence may be exerted by the environment, not only on viral-associated autoreactivity, but also on the immunomodulation of the viral adaptive response. In this review, we have emphasized the importance of the environment, particularly EDCs and estrogen mimicry, and the role played in the disruption of immune dynamics. We show that both innate and adaptive response rely upon hormone transduction for several key functions, including cytokine secretion and proliferation.

EDCs set forth a spectrum of effects and determining how immune populations are affected is constrained by several challenges: (1) dose, (2) dose schedule, (3) biological model, (4) cumulative exposure, and (5) metabolites. For many EDCs, dose, including frequency and duration is a critical factor, especially as it may mask biphasic EDC activities. The choice of biological model should also be considered in parallel with dose, as EDCs can produce dramatically different results across species. This variability is likely characteristic of circulating hormone levels and the degree of endocrine involvement, both of which should be taken into account whenever studies are designed, or findings are inferred to human health. In an effort to address this, we hope our review will provide a summary to direct comparisons across both the dose and biological model.

Furthermore, we caution against experimental oversimplification. Although fewer conditions generally allow for clear and translatable interpretations, this may also result in a context that is too artificial to recapitulate the biological phenomenon. Current EDC studies have not adequately addressed effects of cumulative exposure, despite the regular occurrence for this in our environment. Evaluation of effects for combined EDCs can reveal critical information to help identify mechanisms and risks of disease onset that are more relevant to natural exposure. EDC metabolites are similarly important in this context of cumulative exposure. Both the inherent ability to metabolize EDCs and the rate at which this occurs warrant consideration and may be useful to resolve the variation of results observed across species and cell types. To aid in this, we have generated mechanism-of-action guides that describe how different categories of environmental endocrine disruptors, including their metabolites, are most likely to interfere in immune-endocrine signaling.

In writing this review, we hope the information herein detailed will remove some of the complexities associated with inferring experimental findings of EDC exposure to human health and may be used as a guide for future research.
